# Diagnosis and management of extramedullary plasmacytoma in nasal cavity: Clinical experience and literature review

**DOI:** 10.1097/MD.0000000000032647

**Published:** 2023-01-13

**Authors:** Hongyu Hu, Xianwen Hu, Guomei Hu, Dandan Li, Jiong Cai

**Affiliations:** a Department of Nuclear Medicine, Affiliated Hospital of Zunyi Medical University, Zunyi, China; b Department of Pathology, The First People’s Hospital of Zunyi City, The Third Affiliated Hospital of Zunyi Medical University, Zunyi, China; c Department of Obstetrics, Zunyi Hospital of Traditional Chinese Medicine, Zunyi, China.

**Keywords:** computed tomography (CT), extramedullary plasmacytoma (EMP), magnetic resonance imaging (MRI), nasal cavity, positron-emission computed tomography (PET)

## Abstract

Nasal extramedullary plasmacytoma (EMP) is a rare plasma cell tumor that occurs in the soft tissue of the nasal cavity, and its imaging characteristics are still unclear. The purpose of this study was to investigate the clinical features, imaging findings, treatment, survival analysis, and prognosis of nasal EMP, and to provide a systematic review of the patients we treated and the published literature. A 45-year-old female patient who presented with epistaxis with nasal obstruction was recommended for magnetic resonance imaging to assess the nature of the lesion. On magnetic resonance imaging, abnormal signal shadow can be seen in the right nasal cavity. Diffusion weighted imaging showed signal of the lesion was significantly limited, presenting high signal, with a low apparent dispersion coefficient, and the lesion was significantly enhanced on contrast-enhanced scan. Combined with the clinical manifestations of the patient, who was initially considered to have a hemangioma. She underwent endoscopic nasal surgery under general anesthesia to remove the mass, and the final pathology confirmed it was EMP. However, the final pathology confirmed EMP. Five months later, the patient came to our hospital for follow-up and underwent fluorine-18-fluorodeoxyglucose/positron emission tomography/computed tomography scan, which showed no recurrence of the lesion and no transformation of multiple myeloma. The nasal EMP imaging findings were mostly soft tissue masses with uniform density or signal, which were significantly enhanced by enhancement scan, high signal on diffusion weighted imaging and low signal on apparent dispersion coefficient. Immunohistochemical staining for CD38, CD138, and CD79a was positive in most of the cases evaluated, while CD20 and CD10 were negative. The absence of dilated features, infiltrative features and the presence of significant contrast enhancement may be relatively specific imaging findings of nasal EMP. The prognosis of nasal EMP is good, and recurrence, metastasis, and transformation into multiple myeloma are rare. Because the lesions are sensitive to radiotherapy, surgical resection combined with radiotherapy is a more effective treatment.

## 1. Introduction

Extramedullary plasmacytoma (EMP) is a rare plasma cell tumor that occurs in soft tissues outside the bone marrow.^[1]^ Compared with other malignant plasma cell diseases, it accounts for only 3% of plasma cell tumors and 1% of head and neck tumors.^[2,3]^ The etiology of these lesions is unknown, but viral pathogenesis and chronic irritation have been suggested as contributing factors.^[4,5]^ A previous study has shown that the incidence of nasal EMPs in men and women is 3:1, and the onset age is usually between 40 and 70 years.^[2]^ The disease often presented with insidious and nonspecific symptoms including epistaxis on the same side of the tumor, rhinorrhea, sore throat, dysphonia, and hemoptysis, which delayed the timely diagnosis of the tumor.^[6]^ Moreover, due to its rarity, the imaging findings of nasal EMP are still unclear. However, computed tomography (CT) may be helpful for adequate local staging of EMP, which can help to observe the size and location of the mass and determine the site of tumor invasion. Although magnetic resonance imaging (MRI) is less sensitive than CT in detecting osteolytic lesions, functional MRI techniques, such as diffusion weighted imaging (DWI), can reflect complex tissue components and provide a better representation of physiological processes, and its use can improve the ability of MRI to distinguish active from inactive lesions.^[7–9]^ DWI is a well-known noninvasive technique to obtain tissue microstructure information by measuring water diffusivity in various tumors and tissues.^[10]^ On DWI, extramedullary plasma cells can show limited diffusion, indicating high cellularity in the lesion.^[11]^ Diffusion-weighted MRI can be evaluated by means of apparent dispersion coefficient (ADC) values, which reflect both water diffusion (random movement of water molecules) and tissue perfusion (microcirculation of blood in a capillary network) in biological tissues. In addition, previous studies have found that malignant sinus lesions have significantly lower ADC values than benign lesions.^[12–14]^ Typically, malignant tumors are limited in their aqueous spread due to their supercellular structure and enlarged nuclei. Thus, low ADC values in malignant tissues can be attributed to increased cells, intracellular distance limitation, and water diffusion limitation. Studies have shown that ADC values are related to cell density and mass secretory properties.^[15]^ Nasal EMP is highly sensitive to radiation, and local surgery combined with radiotherapy is effective, the prognosis of which depends on the size of the tumor and lymph node involvement, and the 10-year survival rate is 50 to 80%.^[16]^ Here, we report a 45-year-old female patient with a right lesion showing slightly homogeneous signal intensity on MRI and significantly enhanced on contrast-enhanced scan, which was considered to be a hemangioma, but was eventually confirmed by pathology to be an EMP of the right nasal cavity. EMP of the nasal cavity is a rare tumor, and most of the current literature on the tumor is in the form of case reports or small-scale retrospective clinical analyses. Therefore, we reviewed the published literature on EMP of the nasal cavity and summarized the epidemiology, imaging and clinical features of the tumor, optimal management and long-term prognosis, and the imaging differential diagnosis was discussed in detail.

## 2. Case presentation

**Figure 1.**
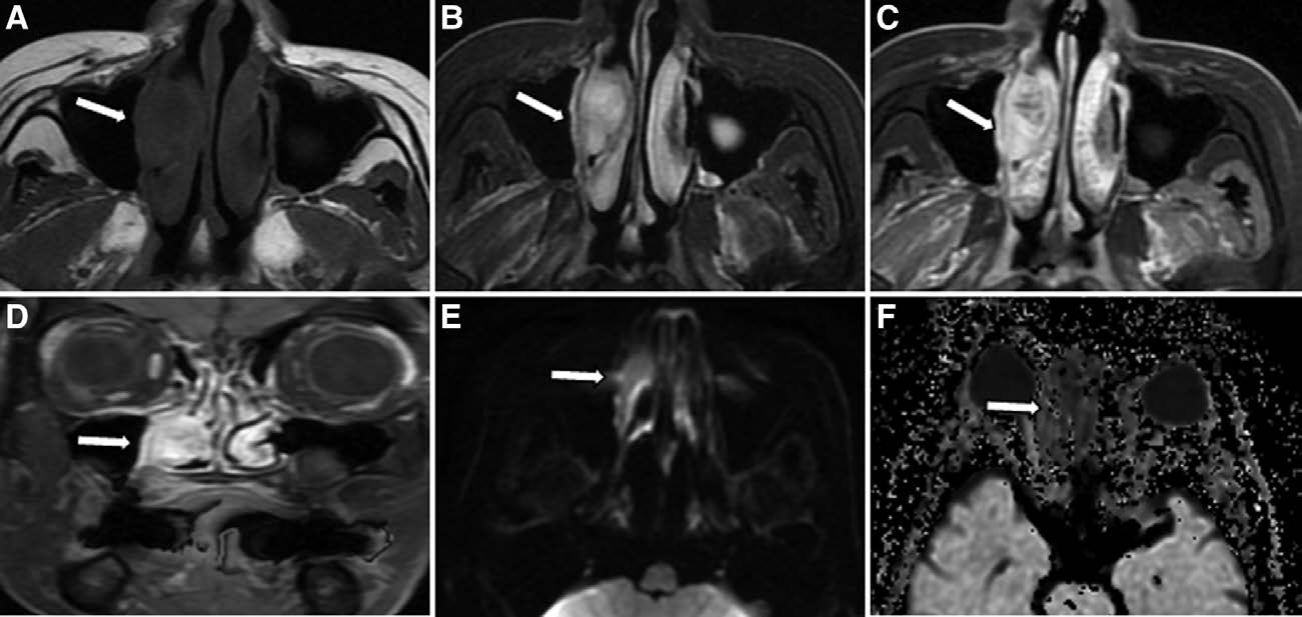
MRI showed that the tumor was located in the right nasal cavity. (A, arrow) T1WI sequence showed that the lesion was hypo-to-isosignal, about 2.7 cm × 1.6 cm in size. (B, arrow) T2WI sequence shows hypersignal. (C, axial, D, coronal, arrows) Contrast-enhanced T1WI showed obvious enhancement of the lesion and dilatation of the surrounding bone. (E, arrow) DWI showed limited spread of the lesion, showing high signal and (F, arrow) a low ADC. ADC = apparent diffusion coefficient, DWI = diffusion weighted imaging, MRI = magnetic resonance imaging, T1WI = T1-weighted imaging.

**Figure 2.**
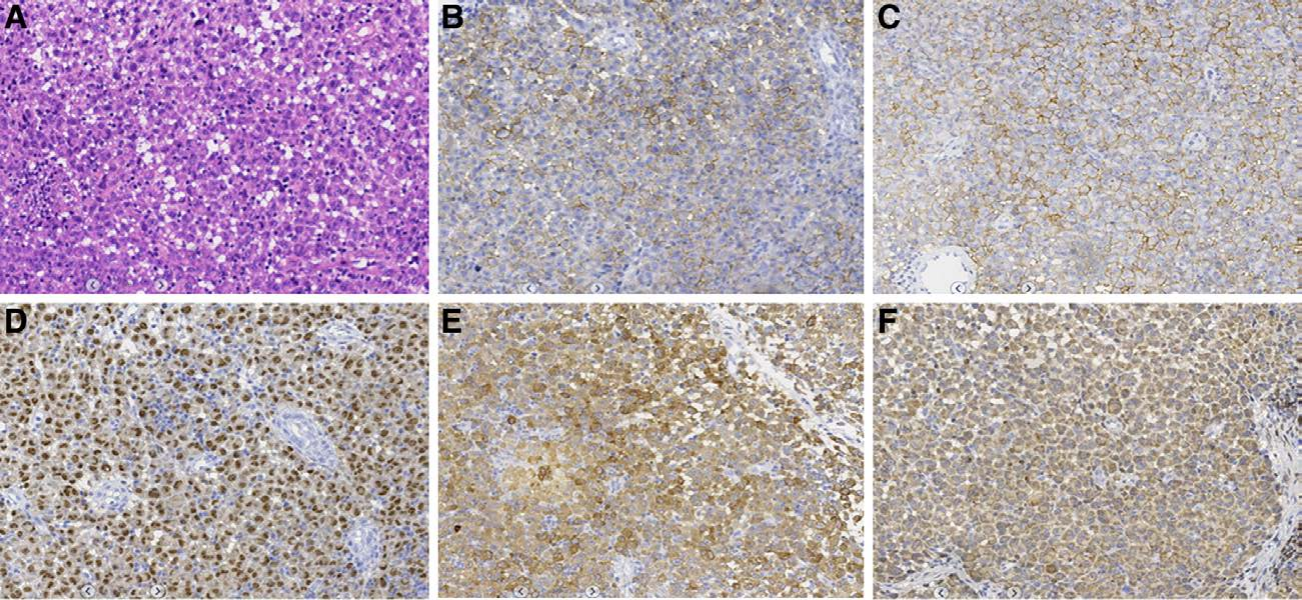
(A) Hematoxylin-eosin staining showed that the tumor cells were diffusely distributed, round, uniform in size, rich in cytoplasm and biased in nucleus. Immunohistochemical staining showed that tumor cells positively expressed (B) CD138, (C) CD38, (D) MUM-1, (E) CD79 and (F) Bcl-2.

A 45-year-old female patient was admitted to the hospital with a 2-month history of epistaxis, which was aggravated and accompanied by nasal congestion for 15 days. Two months ago, the patient developed epistaxis, cough, dizziness, headache, and hyposmia without obvious triggers. Fifteen days before the onset of the disease, the above symptoms became significantly worse, accompanied by nasal congestion, no nasal itching, nasal pain and other discomfort. No symptomatic treatment of the disease was received during this period. His father and brother both suffered from malignant tumors. His father died of lung cancer, and his brother was still alive after treatment for rectal cancer, while other family members had no history of malignant tumors. Physical examination showed no external nasal collapse, no nasal stenosis, chronic congestion of bilateral nasal mucosa, red discharge from the right nasal cavity, with mass filling and easy bleeding to touch. No significant abnormalities were found in the left nasal cavity. It is recommended that the patient undergo MRI to evaluate the nature of the lesion. On MRI, the lesion is isohyintense on T1-weighted imaging (T1WI) and homogeneous hyperintense on T2WI. The size of the lesion is about 2.7 cm × 1.6 cm, and the mass is significantly enhanced on contrast-enhanced scan (as shown in Fig. 1). According to the above imaging findings, she was suspected to be a hemangioma. Subsequently, endoscopic examination revealed a pink neoplasm in the right nasal cavity with blood crust and pseudomembrane on the surface, enlargement of the maxillary sinus and a small amount of sinus fluid. In addition, some new organisms were seen in the sinus mucosa. The patient underwent “endoscopic resection of the right nasal mass” under general anesthesia. Pathological examination showed that the tumor cells were round, uniform in size, rich in cytoplasm, and skewed in nucleus. Immunohistochemical results showed that tumor cells expressed CD138+, CD38+, MUM-1+, CD79a, BCL-2 and *κ* light chains (as shown in Fig. 2). Based on these features, the pathologist diagnosed the patient with EMP in the right nasal cavity. She was then given 52 Gy dose of local radiotherapy in the right nasal cavity. To complete the diagnostic process, the patient underwent hematologic screening, protein electrophoresis, Bence Jones proteinuria, and bone marrow analysis, all of which showed no abnormalities. While most EMP cases are not immediately life-threatening at diagnosis, EMP can progress to multiple myeloma (MM) and therefore requires monitoring. In order to evaluate the condition of the lesion in the surgical area and the systemic disease, the patient came to our hospital for follow-up 5 months later and underwent fluorine-18-fluorodeoxyglucose (^18^F-FDG)/positron emission CT (PET)/CT scan, which showed no recurrence of the lesion and no transformation of MM.

## 3. Literature review

The PubMed, Embase, and Web of Science databases were searched for case reports and case series of nasal EMP published from October 1, 2000 to October 1, 2022, with language restrictions limited to English. The following search strategy was used: (Nasal Cavity OR Nose) AND (Extramedullary Plasmacytoma OR Extramedullary Plasma Cell Tumor). The first author, year of publication, and country of each case, along with the patient’s age, gender, mass size, side of onset, major clinical symptoms, CT, MRI, or PET/CT imaging results, immunohistochemical results, treatment methods, and follow-up duration and results were extracted (as shown in Table 1).

**Table 1:** Clinical and imaging features of extramedullary plasmacytoma of the nasal cavity in the literature review.

Case, no.	Author, yr, country	Gender/Age	Follow-up (mo)	History	Size (cm)	Location, (L, R, B)	Expansion of peripheral bone	CT NE/CE	MRI (T1WI/T2WI/CE)	PET uptake value	IHC	Recurrence	Metastases at diagnosis	MM	Treatment
1	Urso L, 2021, Italy^[9]^	M/60	–	Papillary thyroid cancer, nasal congestion and rhinolalia	1.7	L	N	Homogeneous isodensity/–	–	12.8	–	–	–	–	RT (50Gy over 25 daily 2Gy)
2	Shrei JA, 2001, Lebanon^[10]^	F/75	Alive/12	Left nasal obstruction and epiphora, left decreased hearing level	–	L	–	Homogeneous isodensity/–	–	–	–	N	N	N	Endoscopic sinus surgery, RT (4400 cGy over a 1-mo period)
3	Shahrizal TA, 2009, Malaysia^[11,17]^	M/54	Alive/–	Right nasal epistaxis, blockage and hyposmia	–	R	–	Homogeneous isodensity/–	–	–	MNFl16, L26, and CD4SRO (UCHLI) (−)	N	N	N	–
4	Windfuhr JP, 2002, Germany^[12]^	M/60	Alive/12	Slightly impaired	–	L	N	–	–	–	CD138, syndecan, CD3 (+), CD20 (−)	N	N	N	RT (55 Gy usually at 2 Gy/d), endoscopic sinus surgery
5	Raghuram S, 2022, India^[13]^	M/45	Alive/29	IgA nephropathy, epistaxis	2.5 × 1.8	L	N	–	Slight hypointense/slight hypointense	–	CD138, MUM-1, lamda, Cyclin D1 (+) CK, CD20, PAX-5, CD3, CD5, CD10, CD23, EBER-ISH (−)	Y	Y	Y	Endoscopic sinus surgery, DT-PACE for 4 cycles.
6	Pantazidou G/2021/Patras^[14]^	M/51	Alive/–	Nasal obstruction	–	L	N	Heterogeneous isodensity/obvious enhancement	–	–	CD79a, CD138, CD56, 30% Ki-67, and vimentin (+)	–	N	Y	RT, chemotherapy
7	Hu X/2022/China^[15]^	M/64	Alive/24	Rhinorrhagia with nasal obstruction	4.2 × 2.5 × 2.0	R	Y	Homogeneous isodensity/–	–	–	CD79a, CD138, CD56, 30% Ki-67, and vimenti (+)	N	N	Y	Endoscopic sinus surgery
8	Hazarika P/2011/India^[2]^	M/31	Alive/5	AIDS was diagnosed 2 mo ago, right nasal obstruction and foul smelling nasal discharge	–	R	Y	Homogeneous isodensity/moderate enhancement	–	–	–	N	N	N	RT (30 Gy)
9	Hazarika P/2011/India^[2]^	M/60	Alive/8	Diabetic and hypertensive, right sided nasal obstruction and recurrent epistaxis	–	R	N	Homogeneous isodensity/moderate enhancement	–	–	–	N	N	N	Endoscopic sinus surgery, RT (30 Gy in ten fractions)
10	Erkal HS/2006/Turkey^[16]^	F/87	Alive/11	Profuse bleeding and difficulty in breathing	–	L	N	Homogeneous isodensity/moderate enhancement	–	–	Cytokeratin, S-100 and CD79a (−) *κ* light chain and epithelial membrane antigen (+)	N	N	N	RT (in daily fractions of 2 Gy for a total dose of 50 Gy).
11	Corvo MA/2013/Sao Paulo^[18]^	F/51	Alive/72	Progressive nasal obstruction, history of smoking and social drinking	2.0 × 1.0	B	Y	Homogeneous isodensity/–	–	–	*κ* chain clonality (+)	N	N	Y	RT, surgery
12	Cheng L/2021/China^[19]^	M/23	Alive/12	ITP, Epstein–Barr virus (EBV)-positive, nasal congestion	–	R	–	–	Slight hypointense/hyperintense/obvious enhancement	–	CD20 (−), CD56 (−), CD3 (−), CD10 (−), bcl-6 (−), CD79a (+), CD99 (+), CD38 (+), CD138 (−), CD5 (−), MUM-1(+), *κ* (−), *λ* (+), and ki-67 (20%+)	Y	Y	Y	CHOP (2-course), PAD (3-course)
13	Belić B/2013/Serbia^[20]^	M/44	Alive/36	Breathing difficulties, right nasal epistaxis	3.5 × 2.0 × 1.5	R	Y	Homogeneous isodensity/obvious enhancement	–	–	CD79a, MUM-1, CD138, CD38 and *λ* (+) Ae1/Ae3, CD20 (−)	N	N	N	Endoscopic sinus surgery, RT (dose of 50 Gray in 25 fractions)
14	Attanasio G/2006/Italy^[21]^	F/67	Alive/12	Smoking habit, hypertension, uterine fibroma, and postoperative breast cancer, unilateral nasal obstruction, discharge, bleeding	–	L	–	Homogeneous isodensity/–	–	–	CD79a, *λ* (+)	N	N	N	Surgery, RT (dose of 40 Gray in 20 fractions)
15	Ashraf MJ/2013/Iran^[22]^	M/20	Alive/12	–	–	L	N	Homogeneous isodensity/–	–	–	–	N	N	N	Endoscopic sinus surgery
16	Ashraf MJ/2013/Iran^[22]^	M/48	Alive/12	Epistaxis	–	L	Y	Homogeneous isodensity/–	–	–	CD38 (+)	N	N	N	Endoscopic sinus surgery, RT (4400 cGy over a 1-mo period).
17	Ashraf MJ/2013/ Iran^[22]^	M/60	Alive/36	Right nasal epistaxis	–	R	–	–	–	–	CD38, EMA (+) r LCA, CD3, CD20 and CK (Dako, Denmark) (−)	Y	N	N	RT
18	Micozkadioglu SD/2009/Turkey^[23]^	F/70	Alive/2	Hypertension, nasal obstruction	3.0 × 1.5	R	–	Homogeneous isodensity/–	–	–	CD38, *κ* light chain (+) *λ* light chain (−)	N	N	N	Endoscopic sinus surgery
19	Lomeo PE/2007/USA^[5]^	F/32	Alive/60	Right nasal congestion with epistaxis	–	R	–	Homogeneous isodensity/–	–	–	–	N	N	N	Endoscopic sinus surgery, RT
20	Xing MH/2021/USA^[24]^	M/70	–	Left nasal obstruction	–	L	–	Homogeneous isodensity/–	Slight hypointense/slight hypointense/obvious enhancement	19.6	CD138, Myc, CD56, p63 (+) MUM2, L20, CD38, CD30, EBER (−)	–	N	N	RT (5-wk course)
21	Adoga AA/2020/Nigeria^[25]^	F/28	Alive/14	Left nasal bleeding with epistaxis		L	Y	Homogeneous isodensity/obvious enhancement	–	–	CD10, CD38 (+)	N	–	–	Endoscopic sinus surgery, RT
22	Ching AS/2002/Singapore^[7]^	M//63	Alive/10	Nasal blockage	–	B	Y	Homogeneous isodensity/moderately contrast	Slight hypointense/slight hypointense/moderate inhomogeneous enhancement	–	–	Y	–	–	RT
	Padhi P/2020/USA^[26]^	M/75	Alive/6	NASAL blockage	3.1 × 4.4 × 3.2	L	Y	Homogeneous isodensity/	–	–	CD38, CD138, CD79a, MUM-1, *κ*, IgA, BCL-2, CD43, ISH, ALK-1, HHV8, *λ*, IgG, IgM, IgD, CD56, CD5, CD10, BCL-6, BCL-1, CAM5.2/AE, CD117, Ki-67 (+) CD20, PAX-5, CD45, CD30, EMA, EBER (−)	N	N	N	RT (40 Gy)

– = not provided, (−) = negative, (+) = positive, B = bilateral, CE = contrast-enhanced, CHOP = cyclophosphamide, doxorubicin, vincristine, prednisone, CT = computed tomography, DT-PACE = dexamethasone, thalidomide, carboplatin, adriamycin, cyclophosphamide, etoposide, F = female, IHC = immunohistochemistry, ITP = idiopathic thrombocytopenic purpura, L = left, M = male, MM = multiple myeloma, MRI = magnetic resonance imaging, N = not, PAD = bortezomib, doxorubicin, dexamethasone, PET = positron emission tomography, R = right, RT = radiotherapy, T1WI = T1-weighted imaging, Y = yes.

**Figure 3.**
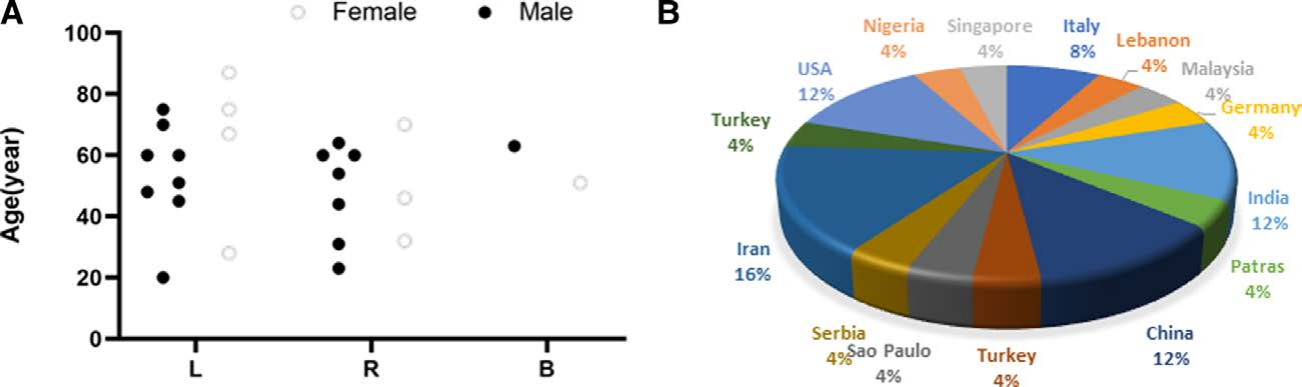
(A) The gender, diseased side and age distribution and (B) country proportion of 23 patients with EMP. B = bilateral, EMP = extramedullary plasmacytoma, L = left, R = right.

**Figure 4.**
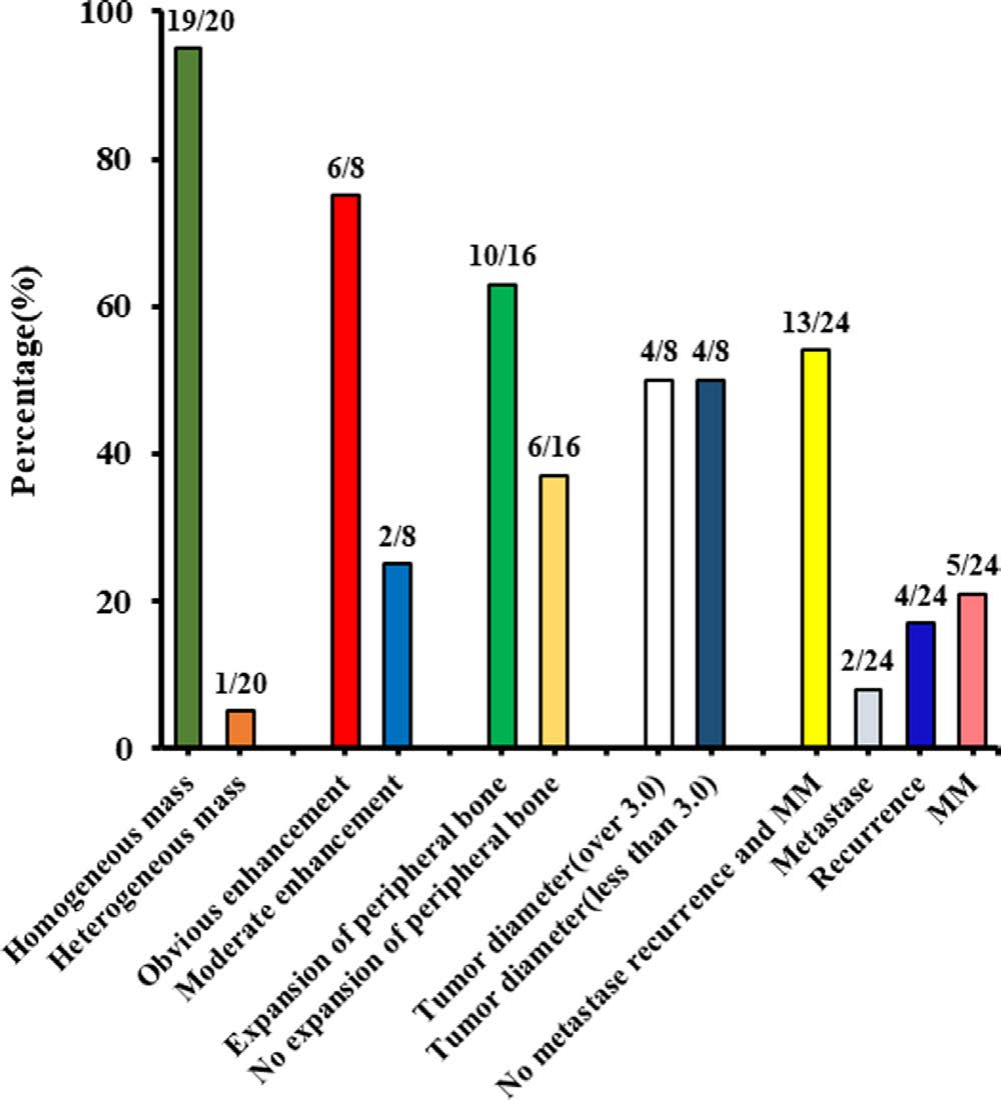
Imaging features of EMP in the nasal cavity. Most of the lesions were soft tissue masses with uniform density or signal, and the lesions showed obvious enhancement on enhanced scan. Most of the lesions showed dilated changes in peripheral bone, and there was no significant difference between lesions > or <3 cm. Metastasis, recurrence, and conversion to MM occurred in a subset of patients with nasal EMP. EMP = extramedullary plasmacytoma, MM = multiple myeloma.

Through systematic and gradual screening of literature, 20 full texts including 23 patients were finally obtained.^[2,5,18–32]^ Including our patient, a total of 24 patients consisting of 16 men and 8 women, were included in this study. The median age of onset in men was about 52.9 years, and the age ranged from 45 to 63 years. The age of onset in females ranged from 51 to 64 years, with a median age of 55.4 years. The top 3 published cases are Iran (16%), India (12%), and China (12%), and the detailed distribution of patients is shown in Figure 3. Only 2 of 24 patients had bilateral nasal tumor infiltration, and there was no significant difference in the incidence of left and right nasal cavity (*P* > .05). The clinical symptoms of 24 patients with nasal EMP were mostly nasal bleeding, nasal congestion, and dyspnea, only a small number of patients had HIV, EBV virus infection history and malignant tumor radiotherapy and chemotherapy history. The imaging findings of EMP in the nasal cavity are mostly soft tissue masses with uniform density or signal, along with an obvious enhancement on contrast-enhanced scan, and the difference in tumor size is not significant. A portion of nasal EMP showed metastasis, recurrence, and progression to MM. Detailed clinical and imaging data are shown in Figure 4. Except for 2 patients who were inoperable due to old age, all the other patients underwent endoscopic tumor resection and radiotherapy. Among them, 1 patient had a history of human immunodeficiency virus infection, 1 patient had a history of idiopathic thrombocytopenic purpura, and 3 patients with MM conversion received chemotherapy. Overall, the prognosis of EMP is good, with 2 patients still alive and healthy at >5 years of follow-up. The immunohistochemistry of nasal EMP was studied and revealed that CD38 (7/8), CD138 (7/8), CD79a (6/7) were positive, and the positive rate were 88%, 88%, and 86%, respectively. Moreover, among the available data, including 3 cases of MUM-1, *κ* light chain were positive, and 5 cases of CD20 and 2 cases of CD10 were negative (Table 2).

**Table 2:** Immunohistochemistry of CD3, CD38, CD79a, CD138, MUM-1 and *κ* light chain was positive in EMP of nasal cavity. Including CD3 positive (1/4), CD38 positive (7/8), CD79a positive (6/7), CD138 positive (7/8), MUM-1 positive (4/4), *κ* light chain positive (4/5), CD10 negative 2 cases and CD20 negative 5 cases.

Marker	Positive cases	Percent (%)
CD3	1 of 4	25
CD38	7 of 8	88
CD79a	6 of 7	86
CD138	7 of 8	88
MUM-1	4 of 4	100
*κ* light chain	4 of 5	80

EMP = extramedullary plasmacytoma, MUM-1 = multiple myeloma oncogenic protein.

## 4. Discussion

Plasmacytoma is a malignant tumor characterized by abnormal monoclonal proliferation of plasma cells that originate in bone (solitary bone plasmacytoma) or soft tissue (EMP).^[33]^ These 2 diseases are extremely rare, with incidence rates of 0.45 and 0.09 per 100,000 people, respectively.^[34]^ The etiology of these lesions is unknown, but viral pathogenesis and chronic irritation have been reported as contributing factors.^[1]^ The incidence of nasal EMP in males is significantly higher than that in females, with the development of the mass, which will have a huge impact on the peripheral nerves, blood vessels or bone damage and cause the corresponding clinical symptoms, including nasal obstruction, bleeding and pain, without specificity. Nasal EMP is a destructive tumor that, in addition to its propensity for local recurrence, has the ability to spread to regional lymph nodes, to metastasize to distant organs and tissues and develop to MM.^[35]^

CT and MRI can be used to assess the local extent and severity of EMP in the nasal cavity, and MRI is superior to CT because of its high soft tissue contrast and multiplanar description, so MRI is recommended as a routine staging for EMP examination.^[36]^ Like most malignant tumors, EMP on MRI is hypointense on T1-weighted imaging and hyperintense on T2-weighted imaging. Some characteristic findings of nasal EMP on MRI have been identified, such as the presence of dilated features, the absence of infiltrative features and the presence of significant contrast enhancement.^[37]^ Diffusion was significantly limited on DWI, and ADC values were significantly lower than those of benign lesions and epithelial-derived malignancies, which contributed to the differential diagnosis. The MRI findings of our patient showed homogeneous signal on T1WI and T2WI, the tumor was significantly enhanced on contrast-enhanced T1WI, and the diffusion of the lesion was obviously limited on DWI, with a low ADC value, which are consistent with typical features of nasal EMP. ^18^F-FDG PET/CT is a whole-body scan based on glucose metabolism to detect the presence of lesions, which is of great significance for diagnosis and staging of EMP, monitoring the recurrence and progression of EMP, showing special advantages in detecting extramedullary infiltration especially.^[17,38]^ Nasal EMP may appear metabolically active on ^18^F-FDG PET/CT with a median maximum standardized uptake value uptake of 5.75.^[39,40]^ However, despite this, the image features of nasal EMP are not specific. According to its imaging manifestations, nasal EMP should be differentiated from some benign lesions such as nasal angiofibroma, nasal polyps and malignant tumors including MM, natural killer/T lymphoma, and cancer of nasal cavity. Nasal angiofibroma is more common in young men, with a history of multiple epistaxis, tumor necrosis is rare, and enhanced scan shows obvious enhancement. The adjacent bone is compressive bone resorption and destruction, and most of the bone deformation.^[41]^ Nasal polyps are mostly soft tissue masses with limited scope, clear boundary, visible tumor pedicle, filled with soft tissue shadow in the nasal cavity, with or without bone resorption and destruction, and most of them are not enhanced after enhancement. On MRI, nasal carcinoma showed iso-signal on T1WI and iso-signal or hypo- signal on T2WI. When the tumor was large, there were necrotic areas with long T1 and long T2 signals, which showed mild or moderate uneven enhancement after enhanced scanning, with no enhanced necrotic liquefaction area. CT showed enlargement of the nasal cavity and obvious lytic bone destruction of adjacent bones. Natural killer/T lymphoma showed homogeneous T1 and T2 signals on MRI, with mild to moderate homogeneous enhancement after enhancement. The CT findings showed that there was no bone destruction when the tumor was small, and bone destruction due to bone remodeling deformation and bone erosion could be seen when the tumor was large.^[24]^ Generally, there was no lytic bone destruction. On MRI, primary malignant melanoma of the nasal cavity shows high signal on T1WI, equal or slightly lower signal on T2WI, and mild to moderate enhancement due to the rich melanin in the tumor can shorten T1 and T2, which is different from the signal performance of other benign and malignant tumors of the nasal cavity.

The accurate diagnosis of nasal EMP still depends on pathological and immunohistochemical evidence. On histopathological examination, monotonous dense plasma cells with round to oval nuclei, vesicular nuclear chromatin, and nuclei often eccentric to the cytoplasm are often seen.^[42]^ Plasmacytoma is characterized by a homogeneous infiltration of monoclonal plasma cells, usually expressing CD138 and/or CD38.^[9]^ When a diagnosis of plasmacytoma is made, follow-up investigations including bone marrow evaluation, urine/serum protein electrophoresis, serum electrophoresis, blood cell count, blood calcium, measurement of renal function, and skeletal examination should be performed to rule out MM (9). Cytogenetic profiles of plasmacytomas in bone marrow samples should be evaluated with fluorescence in situ hybridization because t (11; 14), t (4; 14), del (13q) and 1q gain were associated with recurrence of EMP and prognosis with MM.^[22]^ Our case had no bone marrow fluorescence in situ hybridization information, so we did not assess the nasal plasma cell tumor cell genetics spectrum, this may affect the potential treatment decisions and it is the limitations of our case study. Endoscopic nasal surgery is a relatively safe and minimally invasive technique that provides sufficient and rapid delivery of high-quality tissue samples for a definitive diagnosis and also provides an effective resection method to remove the tumor from the cavity, which can significantly alleviate symptoms and inhibit the progression of the disease. Alexiou et al reviewed the head and neck EMP in detail and found that the combination of radiation and surgery resulted in a significant lead in 5-year survival, with a 5-year survival rate of approximately 50%.^[23]^ Moreover, another study revealed that 40% of EMPs spread beyond the primary site and/or developed lymphatic drainage, 62% of patients had deposits in soft tissues and internal organs, and 81% of patients developed bone lesions.^[12]^ The results of our study on our patient and previous literature review show that nasal EMP has less progression to MM, recurrence and metastasis, which may be related to the short follow-up time.

In conclusion, nasal cavity EMP is usually uniform low signal on T1WI and high signal on T2WI. The diffusion of lesions on DWI is obviously limited, with a low, and obvious enhancement on contrast-enhanced T1WI. Although the clinical features and imaging findings of nasal EMP are not specific, imaging is still of great significance in revealing the tumor site, surrounding bone destruction and invasion of adjacent important structures, guiding surgery and so on. The prognosis of nasal EMP is good, because the tumor is radiosensitive, so radiotherapy is effective, early radical surgery combined with radiotherapy can achieve satisfactory efficacy. Our study provides a reference for the diagnosis and management of nasal EMP through the experience of our patient and the review of published literature. Future studies with additional cases and long-term follow-up are needed to fully understand nasal EMP.

## Author contributions

**Conceptualization:** Xianwen Hu.

**Data curation:** Guomei Hu.

**Funding acquisition:** Jiong Cai.

**Investigation:** Dandan Li.

**Methodology:** Xianwen Hu, Guomei Hu, Dandan Li.

**Writing – original draft:** Hongyu Hu, Xianwen Hu.

**Writing – review & editing:** Xianwen Hu, Jiong Cai.
